# Overproduction, purification, and characterization of nanosized polyphosphate bodies from *Synechococcus* sp. PCC 7002

**DOI:** 10.1186/s12934-018-0870-6

**Published:** 2018-02-20

**Authors:** Fengzheng Gao, Haohao Wu, Mingyong Zeng, Min Huang, Guangxin Feng

**Affiliations:** College of Food Science and Engineering, Ocean University of China, 5 Yushan Road, Qingdao, 266003 Shandong China

**Keywords:** Polyphosphate bodies, Polyphosphate kinase gene, *Synechococcus* sp. PCC 7002, Human intestinal epithelial cells, Lipid droplets

## Abstract

**Background:**

Inorganic polyphosphate bodies (PPB) have recently been linked to a variety of functions in mammalian cells. To improve the yield of PPB from *Synechococcus* sp. PCC 7002 and characterize its form, in this study, a recombinant plasmid containing a polyphosphate kinase (*ppk*) gene was generated and transformed into *Synechococcus* sp. PCC 7002.

**Results:**

PPB separated by Sephadex G-100 was characterized and added to polarized human intestinal epithelial (Caco-2) cells, and the absorption effect was assessed. The *ppk* gene was stably expressed by induction with 1 μM nickel, and the resulting PPB yield from *Synechococcus* sp. PCC 7002 cells increased by 89.66%. Transmission electron microscopy and dynamic light scattering analyses showed that PPB from these cells were nanosized, ranging from a few to approximately 100 nanometres in diameter. PPB can be taken up by Caco-2 cells and are mainly distributed around lipid droplets.

**Conclusions:**

We determined that PPB can be overproduced in *Synechococcus* sp. PCC 7002 and that the resulting PPB were well absorbed by Caco-2 cells. Microalgae provide a promising “cell factory” for PPB production.

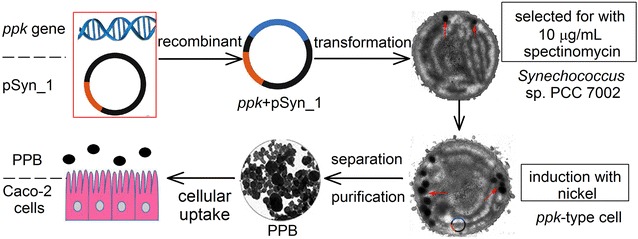

## Background

Most microorganisms, including microalgae [1, 2], have several acclimation strategies to deal with phosphorus limitation and are able to store phosphorus when it is available in excess [3–6]. Inorganic polyphosphate (polyP) is a linear polymer composed of three to several hundred phosphate groups that is found ubiquitously [7]. PolyP plays an important role in supplying raw material for DNA synthesis during the cell division cycle and plays roles in nitrogen fixation and nutrition storage [8, 9]. PPB (also known as volutin granules) are mineralized structures present in many cells and contain large amounts of polyP [10]. Many cells can accumulate PPB, which can then function as a source of phosphate for the synthesis of nucleic acids and phospholipids or be used for ATP synthesis [11, 12].

Mineral nanoparticles have unique advantages in nutrition intervention [13, 14]. Unlike the short-chain polyP used in the food industry, microorganisms synthesize polyP can be hardly processed by alkaline phosphatase in mammalian intestines [15], so that it can be absorbed by the intestinal epithelial cells with endocytosis [16]. PPB play a probiotic role in protecting intestinal tissue from oxidative damage, inflammation, fibrosis and cancerous lesions [17, 18]. As a carrier of mineral elements [19], such as calcium [20], iron [21] and zinc [22], it is possible to develop PPB as a new mineral nutrition enhancer; however, there are no relevant reports on this subject at present.

PPB have been reported to have an important function during the regreening process of several microalgae, including *Anabaena torulosa* [23], *Chlorella vulgaris* [24] and *Synechococcus* 7942. *Synechococcus* is one of the main groups of marine cyanobacteria and it plays an important role in the food chain and carbon cycle [25]. *Synechococcus* sp. PCC 7002 has long been a model organism, alongside *Synechocystis* sp. PCC 6803 and *Synechococcus elongatus* PCC 7942, in studies aimed at understanding photosynthetic prokaryotes [26]. As a naturally transformable organism with a sequenced genome, the strain is an excellent platform for genetic engineering in which many foreign genes have been successfully expressed [27–29]. PPB from *Synechococcus* sp. PCC 7002 are nanosized and much smaller than the PPB obtained from other biological sources and may have special biological activities due to their smaller size and marine origin. However, the ability to obtain high yield of these PPB is one of the main restricting factors for their application. One of the most efficient way to lower the cost for microalgae application is yield improving [30]. The *ppk* gene is widely found in many bacteria and plays an important role in the production of polyP [7, 31, 32]. Overexpression of the *ppk* gene is an efficient way to increase the yield of PPB via the overproduction of polyP [33]. Transmission electron microscopy (TEM) is usually used to observe the appearance of PPB in cells, and the level of PPB can be determined by 4′,6-diamidino-2-phenylindole (DAPI) quantification [34, 35].

*Synechococcus* sp. PCC 7002 is an excellent “cell factory” for PPB production by overexpression of the *ppk* gene. The goal of this study was to improve the yield of PPB from *Synechococcus* sp. PCC 7002 through construction and transformation of a plasmid expressing *ppk* gene into this strain. In addition, we purified and characterized PPB from the engineered *Synechococcus* sp. PCC 7002 and observed their absorption efficiency in Caco-2 cells, with the expectation of PPB having future applications in human health. This study not only is vital for the development of novel nanosized functional particles but also provides a novel method to utilize marine microalgae resources.

## Methods

### Materials

The *Synechococcus* sp. PCC 7002 strain used in this study was kindly provided by Prof. Jindong Zhao from the Institute of Hydrobiology, Chinese Academy of Sciences, and was cultured in Medium A [36] at 32 °C under a light intensity of 100 µmol/m^2^ s. The pSyn_1 vector (3780 bp) and One Shot^®^ TOP10 Chemically Competent *E. coli* were obtained from Invitrogen Corporation. Restriction enzymes (*Hin*dIII and *Kpn*I) and T4 DNA Ligase were obtained from New England Biolabs. The pEASY-Blunt Zero Cloning Kit was obtained from TransGen Biotech Co. Ltd. (Beijing, China).

### DNA manipulations

Chromosomal DNA was isolated from *Synechococcus* sp. PCC 7002 using a Generation Capture Column Kit (QIAGEN). The *ppk* gene sequence was obtained from the NCBI GenBank database (2157 bp, Accession No.: NC_010475.1). Primer sequences:

*ppk* forward primer: CCCAAGCTTGAAGGAGATAAAAAGTATATGTCCTCTGCG;
*ppk* reverse primer: CGGGGTACCCTAATCCAAGCTGTCCTGGAG.

Primer pairs were designed to amplify a ribosome binding site (RBS) as well as *Hin*dIII and *Kpn*I restriction sites at opposing ends of the polymerase chain reaction (PCR) product. The *ppk* gene does not naturally include these two restriction sites, ensuring that the gene itself would not be cut during the digestion. We also ensured that the distance between the *Hin*dIII and *Kpn*I restriction sites in the pSyn_1 vector was sufficient to allow for digestion. The RBS sequence GAAGGAG [37] was placed after the *Hin*dIII restriction site in the forward primer and was strong enough for a maximal level of translation. PCR amplifications were carried out in 50 μL reaction mixtures, and the PCR programme used for amplification was as follows: 5 min at 94 °C; 30 s at 94 °C, 40 s at 60 °C, 1 min at 72 °C for 30 cycles; 10 min at 72 °C. The PCR products were purified and were observed by polyacrylamide gel electrophoresis (PAGE). The DNA polymerase used in the PCR was an ultra-fidelity enzyme to ensure the correctness of the gene amplification. In this study, the target gene was sequenced after every operation step to ensure the correctness.

### Cloning of target gene and plasmid

To obtain enough of the PCR amplified *ppk* gene to be digested with restriction enzymes, the purified target gene was cloned into the pEASY-Blunt Zero vector (3956 bp) at 25 °C for 20 min. The recombinant vector containing the *ppk* gene was named as *ppk*+pEASY. The *ppk*+pEASY and pSyn_1 vector were transformed into TOP10 Chemically Competent *E. coli* for propagation and maintenance. *E. coli* transformants containing the *ppk*+pEASY vector were selected on LB plates with 100 μg/mL ampicillin, while strains containing the pSyn_1 vector were selected for with 100 μg/mL spectinomycin. Single colonies containing the target vectors were cultured in liquid LB medium for approximately 10 h. Plasmids were extracted and purified from *E. coli* using general methods.

### Plasmid construction

The *ppk*+pEASY and pSyn_1 vectors were digested with *Hin*dIII and *Kpn*I restriction enzymes to generate linear DNA fragments with compatible cohesive ends. The digestion products were observed by PAGE. The digested target gene and pSyn_1 vector were recovered and purified. The purified *ppk* insert (1.5 μg) and pSyn_1 vector (150 ng) were ligated together using T4 DNA ligase at 16 °C for 60 min in the provided reaction buffer [50 mM Tris–HCl (pH 7.5), 10 mM MgCl_2_, 10 mM DTT, 1 mM ATP]. The ligation time was extended appropriately if the ligation efficiency was low. The T4 DNA ligase was deactivated at 65 °C for 10 min, and then the ligated products were transformed into TOP10 Chemically Competent *E. coli*. The transformants were selected on LB plates containing 100 μg/mL spectinomycin. The target strain was confirmed to contain the *ppk*+pSyn_1 vector by single-colony PCR. Single colonies selected from the plates were cultured in LB liquid medium. To test the correctness of the constructs, extracted and purified plasmids were digested with either *Hin*dIII or *Kpn*I restriction enzyme or were double digested with both enzymes. The plasmid and digested linear DNA fragments were observed by PAGE. The constructed plasmid was transformed into the TOP 10 *E. coli* strain for propagation and maintenance.

### Transformation of *Synechococcus* sp. PCC 7002

A total of 1.5 mL of *Synechococcus* sp. PCC 7002 (OD_750_ of 1–2) was harvested by centrifugation at 18,630*g* for 3 min at room temperature. The supernatant was removed by pipetting, and the cells were resuspended in 1 mL of fresh medium A by gently pipetting up and down. The cells were centrifuged at 18,630*g* for 1 min at room temperature and suspended with 100 μL of fresh medium A after the supernatant was removed by pipetting. As *Synechococcus* sp. PCC 7002 is competent, exogenous plasmids can be transformed into cells under natural culture conditions. A total of 100 ng of *ppk*+pSyn_1 plasmid DNA was added into the suspended cells. After 24 h of cultivation with weak light, *Synechococcus* sp. PCC 7002 transformants were selected for on medium A plates for 5–7 days with 10 μg/mL spectinomycin. A selected single colony was passaged several times to test of expression stability and was then cultured in 250 mL flasks for plasmid extraction. The purified plasmids extracted from *ppk*-type strains of *Synechococcus* sp. PCC 7002 were used as a template for PCR using the *ppk* primers and were sequenced by The Beijing Genomics Institute.

### Nickel induction and polyP measurements

The neutral site vector pSyn_1 has a nickel activated promoter from *S. elongatus* PCC 6803. Expression was tested using 0.5, 1, 2, or 5 μM Ni^2+^ (in the form of nickel nitrate) added to Medium A. A culture treated without nickel was used as a control. All groups in this experiment had the same inoculation concentration and culture time. *OD*_750_ was measured every day to reflect the growth rate difference of cultures with different concentrations of Ni^2+^. PPB were extracted from *Synechococcus* sp. PCC 7002 by water boiling bath for 10 min with HEPES buffer (20 mM HEPES pH 7.0, 150 mM KCl). Liquid supernatant was obtained by centrifugation at 18,630*g* for 10 min at room temperature. PolyP has fluorescence at 550 nm with DAPI without prior purification and occurs almost without interference from DNA, RNA and ADP. The fluorescence intensity can reflect the PolyP quantification [34].

Fluorescence measurements were performed using a HITACHI F-4600 Fluorescence Spectrofluorometer at an excitation wavelength of 415 nm, electric tension of 700 V and emission wavelength of 550 nm. The excitation bandwidth was 5 nm, and the emission bandwidth was 10 nm. Supernatant samples (500 μL) were prepared in microcentrifuge tubes with HEPES buffer and stained with 60 μL of 100 μΜ DAPI; they were then vortexed for 5 s, incubated for at least 7 min, vortexed again, and then measured in a final volume of 3 mL of HEPES buffer in a quartz fluorescence cuvette.

### PPB purification and characterization

The supernatant of *Synechococcus* sp. PCC 7002 obtained from 10 min boiling was centrifuged [34], concentrated and filtered through 0.22-μm cellulose acetate filter before separation. Filtered supernatant was added to a Sephadex G-100 chromatography column and was eluted with ultrapure water at a 0.5 mL/min flow rate. An aliquot from every corresponding peak was collected and detected with DAPI. The separated samples were concentrated and observed by SDS-PAGE for separation effect analysis. The separated sample with high DAPI fluorescence was filtered through 0.22-μm cellulose acetate filter and observed using TEM and dynamic light scattering (DLS).

A total of 1 mL of wild-type and *ppk*-type *Synechococcus* sp. PCC 7002 cells were harvested by centrifugation at 9500*g* for 1 min at room temperature. The cells were cleaned with medium A and normal saline and then were centrifuged at 9500*g* for 1 min. For conventional electron microscopy, collected cells were fixated with 2.5% glutaraldehyde in 0.1 M phosphate buffer solution (PBS, pH 7.4) for 2 h at room temperature and in a 4 °C refrigerator overnight. After glutaraldehyde fixation, the cells were cleaned with PBS for three times for 15 min respectively. The cleaned cells were post-fixed in 1% OsO_4_ for 1–1.5 h and then were cleaned again as method mentioned above. The cells were dehydrated with 50, 70 and 90% acetone respectively for 15 min, and finally dehydrated with 100% acetone for 20 min for three times. Epon-812 was utilized for resin embedment (100% acetone: embedment solution 2:1 for 30 min; 100% acetone: embedment solution 1:2 for 1.5 h at 37 °C; embedment solution for 2–3 h at 37 °C). The cells were solidified for 24 h at 37, 45 and 60 °C respectively. The ultrathin sections were provided by Reichert-Jung Ultracut E ultramicrotome with thick of 70 nm. After staining with uranyl acetate for 15 min and lead citrate for 10 min, sections were observed with a JEM1200 TEM. The sample used for DLS was added to the formvar stabilized with carbon support films and observed by TEM also.

### Cellular uptake experiment

The polarized Caco-2 cell model is frequently used to simulate human intestinal absorption [38]. Caco-2 cells were obtained from the Cell Bank of the Chinese Academy of Sciences (Shanghai, China) and seeded at a density of 5 × 10^4^ cells/cm^2^ in collagen-treated 24-well plates (BD Biosciences, San Jose, CA, USA). The differentiated cells were gained according to previous method [38] and were cultured in serum-free minimum essential medium (MEM) supplemented with 4 mg/L hydrocortisone, 5 μg/L selenium, 5 mg/L insulin, 34 μg/L triiodothyronine, and 20 μg/L epidermal growth factor for 24 h. A total of 10 μg/mL PPB (final concentration) was added to the MEM cell culture. After 30 min of treatment, Caco-2 cells were washed twice with PBS (10 mM, pH 7.4) and then fixed with 2.5% glutaraldehyde. The ultrathin sections were prepared as the method mentioned above, however the centrifugation parameter should be 800*g* for 10 min for Caco-2 cells. The sections were observed by TEM. Normal cells without PPB treatment were also prepared as ultrathin sections and observed by TEM.

## Results

### Objective gene cloning and purification

**Fig. 1:**
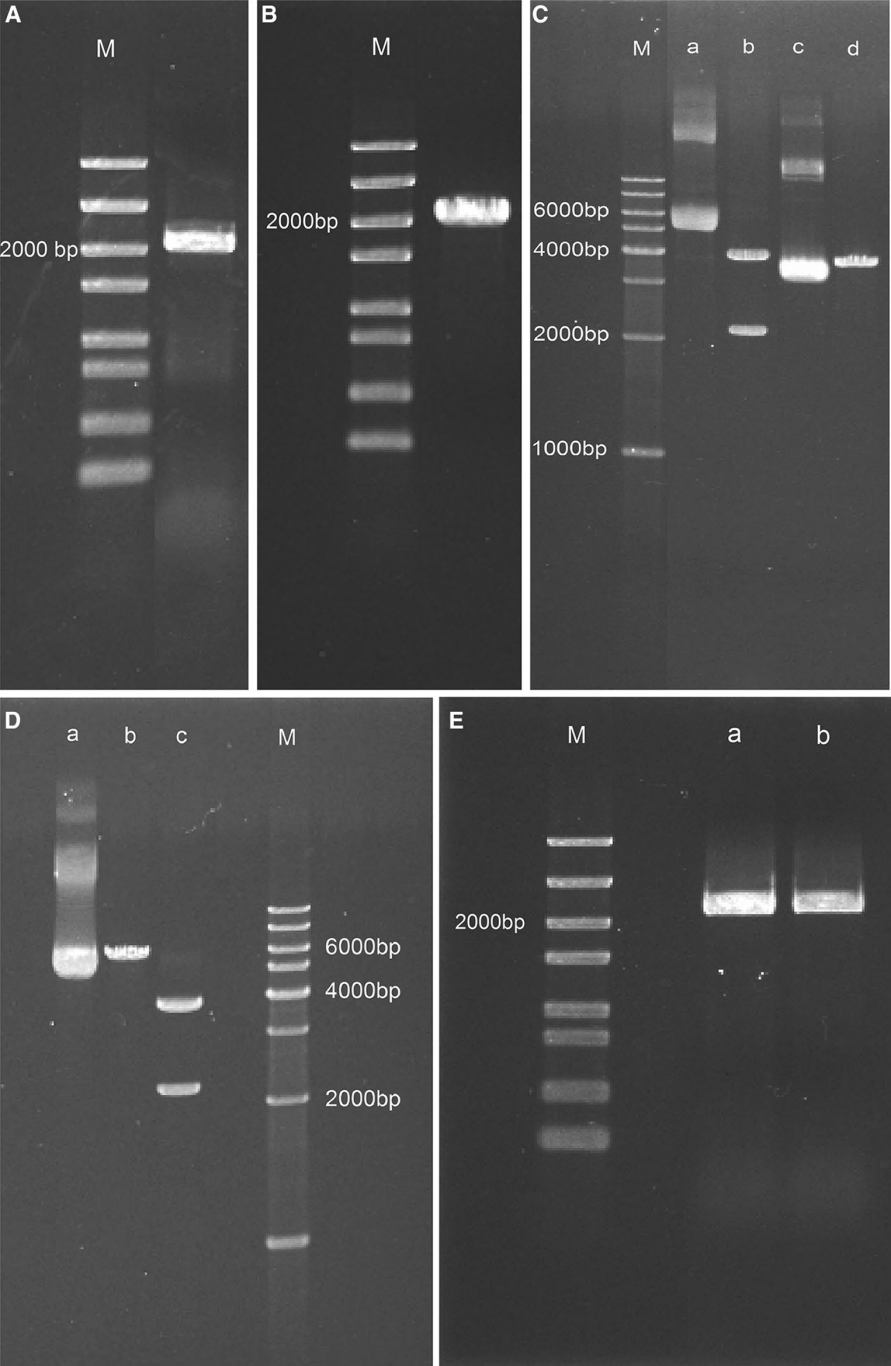
Electrophoresis results of the construction of transgenic strains on a 1% agarose gel. **A** PCR products of target gene; **B** purified target gene from PCR products; **C** lane a: *ppk*+pEASY-Blunt vector; line b: *ppk*+pEASY-Blunt vector digested with *Hin*dIII and *Kpn*I restriction enzymes; line c: pSyn_1 vector; line d: pSyn_1 vector digested with *Hin*dIII and *Kpn*I restriction enzymes; **D** lane a: *ppk*+pSyn_1 vector; line b: *ppk*+pSyn_1 vector digested with *Hin*dIII or *Kpn*I restriction enzymes; line c: *ppk*+pSyn_1 vector digested with *Hin*dIII and *Kpn*I restriction enzymes; **E** PCR products from *ppk*-type strain plasmid (lane a and b); M in **A**, **B**, **E** was 5 Kb DNA Marker, in **C**, **D** was 1 Kb DNA Ladder

The isolated chromosomal DNA from *Synechococcus* sp. PCC 7002 was cloned using *ppk* primers by PCR and the PCR products were observed by PAGE. As shown in Fig. 1A, the target gene (2157 bp) obtained by PCR was slightly larger than 2000 bp on the gel. The *ppk* gene was amplified with a RBS, as well as the restriction enzyme cutting sites *Hin*dIII and *Kpn*I at either end. Faint non-specific bands and primer dimers bands can be seen on the gel. The primers were somewhat long because of the RBS and restriction enzyme cutting sites, which likely increased the possibility non-specific amplification products. To obtain the purified target gene, the PCR products were purified and can be observed clearly in Fig. 1B without interfering bands. The purified target gene was sequenced by Beijing Genomics Institute and was confirmed as the *ppk* gene of *Synechococcus* sp. PCC 7002 without mutation, which is important to the success of the study.

### Objective gene and vector propagation

To reveal the same enzyme cutting sites, *ppk*+pEASY and pSyn_1 vector extracted from TOP 10 *E. coli* cells were digested with *Hin*dIII and *Kpn*I restriction enzymes. The *ppk*+pEASY vector was verified as lane a in Fig. 1C. The electrophoretic band above 6000 bp is concatenated plasmid. Intact supercoiled plasmid is not linear but become so after digestion. Two bands were visible in the *ppk*+pEASY vector digested sample (lane b) with sizes of approximately 2157 bp (target gene) and 3956 bp (pEASY-Blunt vector), respectively. The pSyn_1 vector was loaded in lane c, and the pSyn_1 vector was digested into two bands with the same restriction enzymes. The smaller band (< 30 bp) was too short to be seen on the agarose gel. We can only observe the long band in lane d, which is approximately 3780 bp. The target gene band (2157 bp in lane b) and the pSyn_1 vector in lane d were purified for ligation.

### Construction of recombinant plasmid and transformants selection

We observed the *ppk*+pSyn_1 plasmid in the gel as lane a in Fig. 1D. Concatenated plasmids can also be observed on the gel. To test the correctness of *ppk*+pSyn_1 vector, the vector was single digested with either the *Hin*dIII or *Kpn*I restriction enzyme. The linear plasmid obtained after digestion was approximately 6000 bp (3780 bp pSyn_1 vector plus 2157 bp *ppk* gene) as seen in lane b. The *ppk*+pSyn_1 vector was also digested with both restriction enzymes, producing two bands with sizes of approximately 4000 and 2000 bp, corresponding to the 3780 bp pSyn_1 vector and 2157 bp *ppk* gene, respectively. The experimental results show that the recombinant *ppk*+pSyn_1 vector was successfully built. The *ppk*+pSyn_1 vector was used to transform *Synechococcus* sp. PCC 7002. We obtained *Synechococcus* sp. PCC 7002 transformants with stable expression of the spectinomycin resistance gene, which exhibited stable expression for at least 12 months. Plasmids extracted from *Synechococcus* sp. PCC 7002 were PCR amplified using the *ppk* primers. The PCR products were approximately 2157 bp on an agarose gel (Fig. 1E, lane a, b) and were confirmed as the *ppk* gene of *Synechococcus* sp. PCC 7002.

### Nickel induction and polyP measurements

**Fig. 2:**
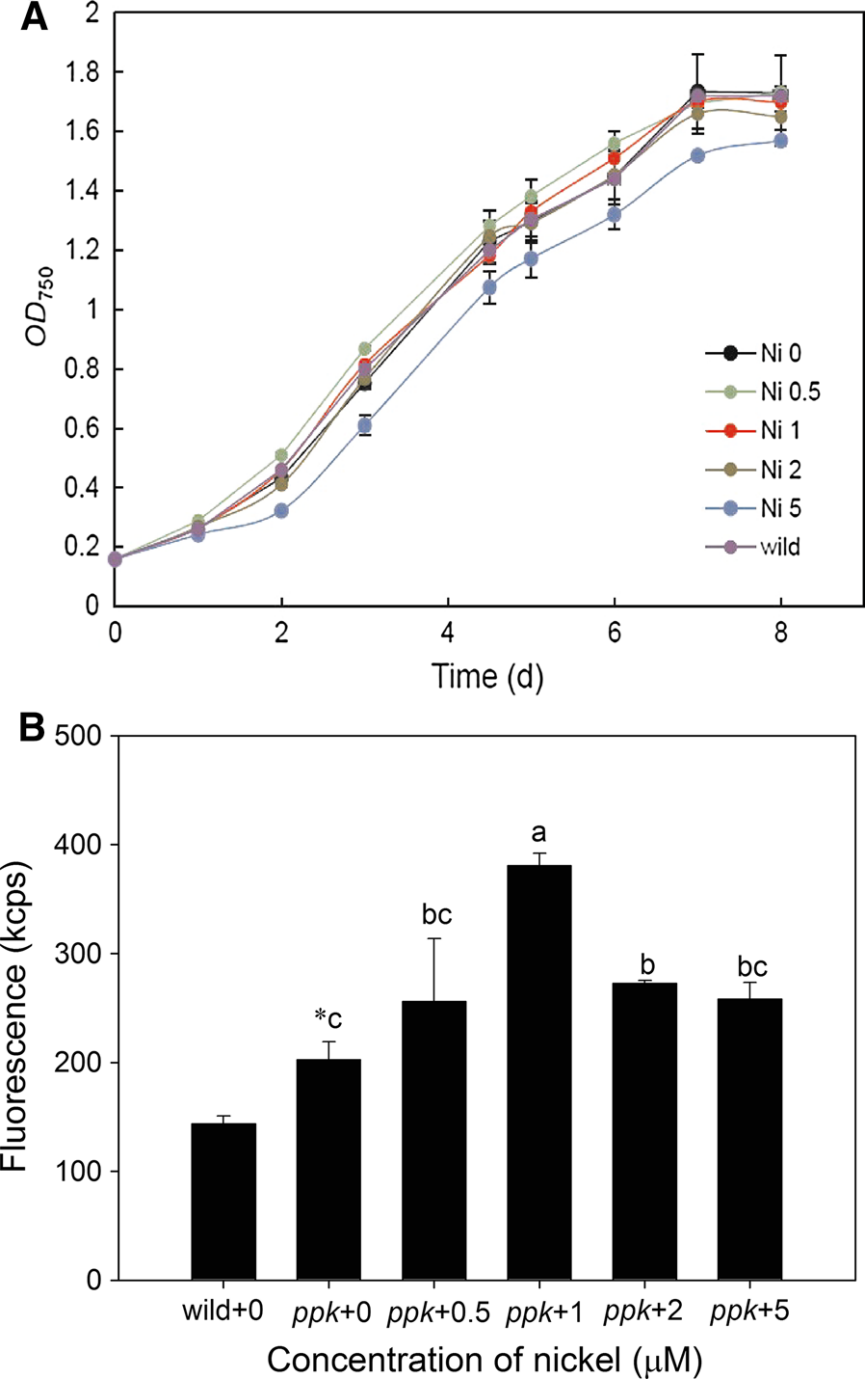
Growth curves and fluorescence intensity of the wild strain and *ppk*-type strain. **A** Growth curves of different cultures of different concentration of Ni^2+^; **B** fluorescence intensity with different concentrations of nickel. Same letters above error bars indicate homogeneous subsets; * indicates significant difference (p < 0.05) between wild-0 group and *ppk*-0 group

The concentration of nickel in culture medium can affect the yield of PPB because of the nickel promoter [39]. The growth curves of different cultures with different concentration of Ni^2+^ are shown as Fig. 2A. There is no significant difference in the growth rate between the *ppk*-type strain cultivated with 0, 0.5, 1, 2 μM Ni^2+^ as well as the wild-type strain cultivated without Ni^2+^. However, 5 μM nickel did not help the cell viability, which indicated toxicity of Ni^2+^ itself. This result was similar with Chen’s report that 2 μM Ni^2+^ did not help the cell viability of the engineering *S. elongatus* PCC7942 [37], while the *ppk*-type cells in this study had a better tolerance with 2 μM nickel.

Fluorescence intensity with different concentrations of nickel treatment is shown in Fig. 2B. The fluorescence intensity of *ppk*-type cells is significantly higher than that of wild-type. In the no-nickel treatment, the fluorescence intensity of the *ppk*-type strain is 40.94% higher than that of wild-type. The fluorescence intensity increased as the nickel concentration increased but decreased when the concentration of nickel rose above 1 μM. The fluorescence intensity was highest with a treatment of 1 μM nickel, 89.66% higher than the wild-type strain and 34.57% higher than the *ppk*-type strain without nickel treatment. An appropriate concentration of nickel can thus induce the expression of the *ppk* gene and then promote the expression of polyP. The result of the overexpression of polyP is the excessive accumulation of PPB. The *ppk*-type *Synechococcus* sp. PCC 7002 strain can produce much more PPB than the wild-type strain because of the overexpression of the *ppk* gene. The result obtained from this research was similar to the result obtained by Chen [37] who observed that 0.5 and 1 μM nickel can improve the cadmium tolerance of *S. elongatus* PCC7942 by *ppk* gene induction.

### PPB purification

**Fig. 3:**
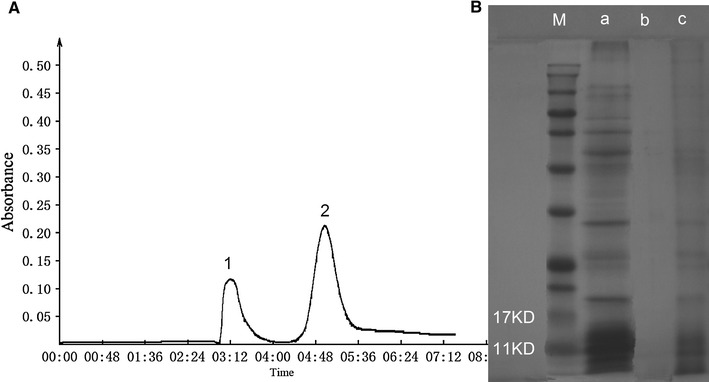
Sephadex G-100 column chromatography of liquid supernatant and SDS-PAGE observation. **A** Sephadex G-100 column chromatography; **B** SDS-PAGE observation; lane a: sample before separation; lane b: sample from peak 1; lane c: sample from peak 2; lane M: marker

PPB from centrifugal supernatant (obtained from 10 min boiling) were separated by Sephadex G-100. The column chromatographic spectrum diagram is shown in Fig. 3A, in which two separated peaks can be observed. The molecular weight of PPB is much larger than that of impurities; thus, the elution speed must be faster than that of other impurities and is very helpful for PPB separation. The sample with the higher DAPI fluorescence from peak 1 in Fig. 3A was PPB, while the other peak was a mixture of compounds (including *Synechococcus* sp. PCC 7002 proteins) with a very low level of DAPI fluorescence.

Filtered supernatant without purification and samples from each separated peak were observed by SDS-PAGE as shown in Fig. 3B. Several protein bands can be observed in lane a (sample before separation) and lane c (sample from peak 2), while no obvious bands were observed in lane b (sample from peak 1) suggesting the proteins and PPB were separated by Sephadex G-100 effectively. The main proteins (in lane a, c), molecular weight ranging from 11 KD to 17 KD, was phycocyanin. The products separated by this method contains a mixture of PPB of different sizes. The PPB separation method using Sephadex G-100 is very simple and convenient. Further purification steps such as ultrafiltration and high-performance liquid chromatography would be needed to obtain PPB with different molecular weights. PPB collected from peak 1 were analysed by DLS and TEM.

### TEM observation and DLS analysis

**Fig. 4:**
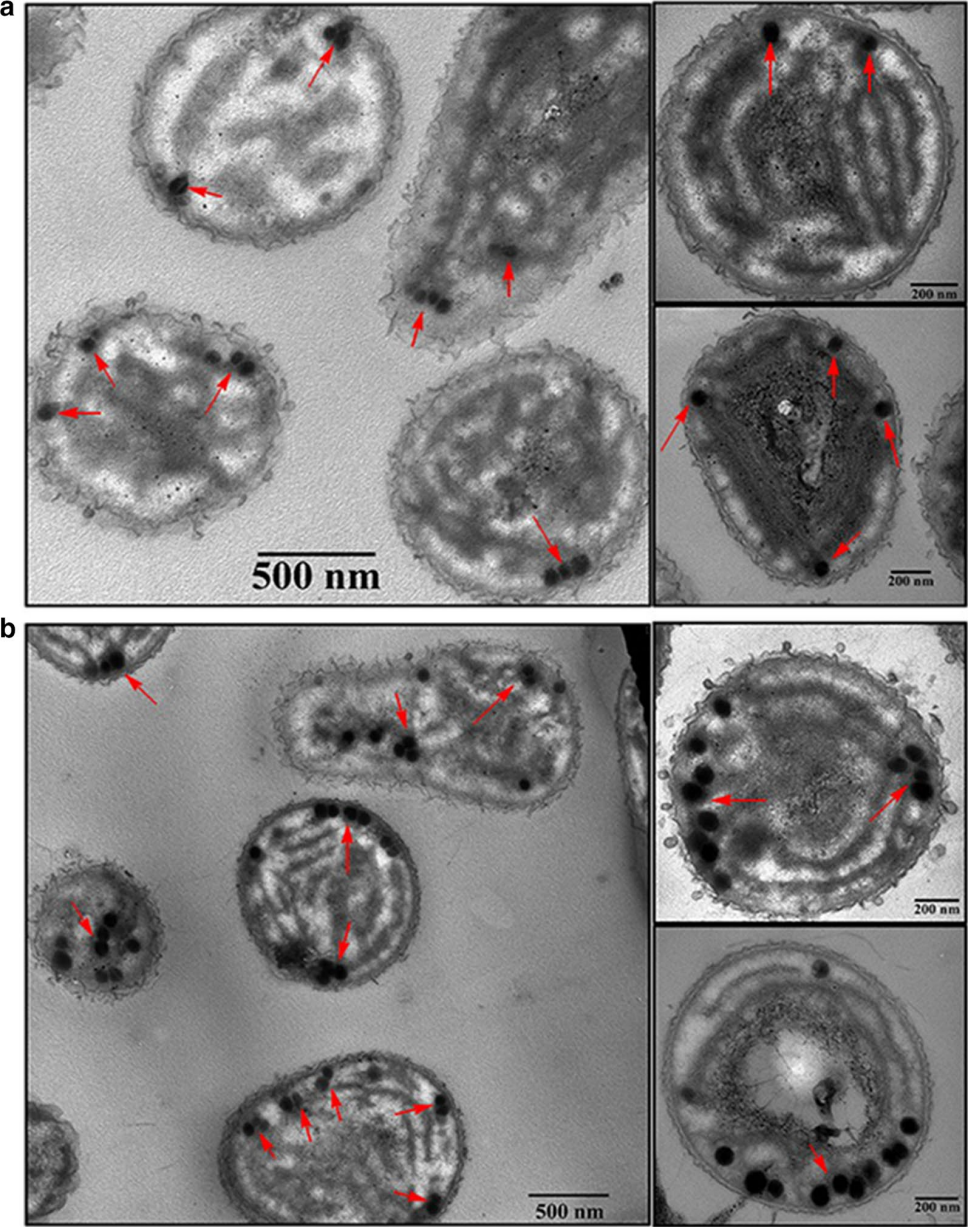
TEM observation of cells. **a** Wild-type cells; **b** *ppk*-type cells

The wild-type and *ppk*-type *Synechococcus* sp. PCC 7002 cells were observed by TEM to clearly observe cellular structures. Because the PPB contained abundant inorganic metal elements, they appeared as clearly observable black spots (Fig. 4). The number of PPB in *ppk*-type cells (Fig. 4b) was much greater than that in the wild-type cells (Fig. 4a). We observed less than 5 PPB in the wild-type cells, while that number was approximately 10 in *ppk*-type cells. This result is in line with the DAPI fluorescence intensity experiment. The number of PPB doubled using the genetic modification approach. The yield of PPB also improved significantly, again indicating that the genetic modification was very successful and likely represents one of the most efficient ways to improve the yield of PPB.

**Fig. 5:**
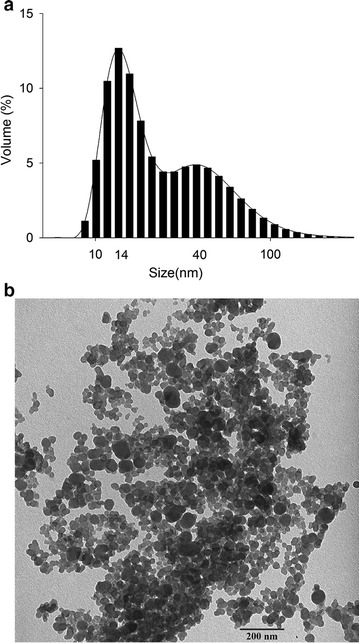
The DLS and TEM results for purified PPB. **a** DLS; **b** TEM

The purified PPB were analysed by DLS and TEM. Figure 5a shows the peaks at 14 and 40 nm that were observed for PPB. The size of PPB ranged from a few to one hundred nanometres and was consistent with the TEM results, though few were larger than 100 nm. In recent years, nanosized materials have become a research hot spot for researchers all around the world. Nanosized PPB from *Synechococcus* sp. PCC 7002 are a new functional material, and the nanosized form improves the application potential for PPB.

The sample analysed by DLS was also observed by TEM. As shown in Fig. 5b, we can clearly see nanosized PPB with different sizes on the photograph. The sizes of almost all the PPB were below 100 nm. The TEM result was consistent with DLS analysis. This result also indicated that PPB kept a good sized form during the boiling extraction process, demonstrating that the thermal stability of PPB is good since they were stable after 10 min of boiling. This characteristic will aid in its further application. The purified PPB were stored at − 20 °C.

### Cellular uptake experiment

**Fig. 6:**
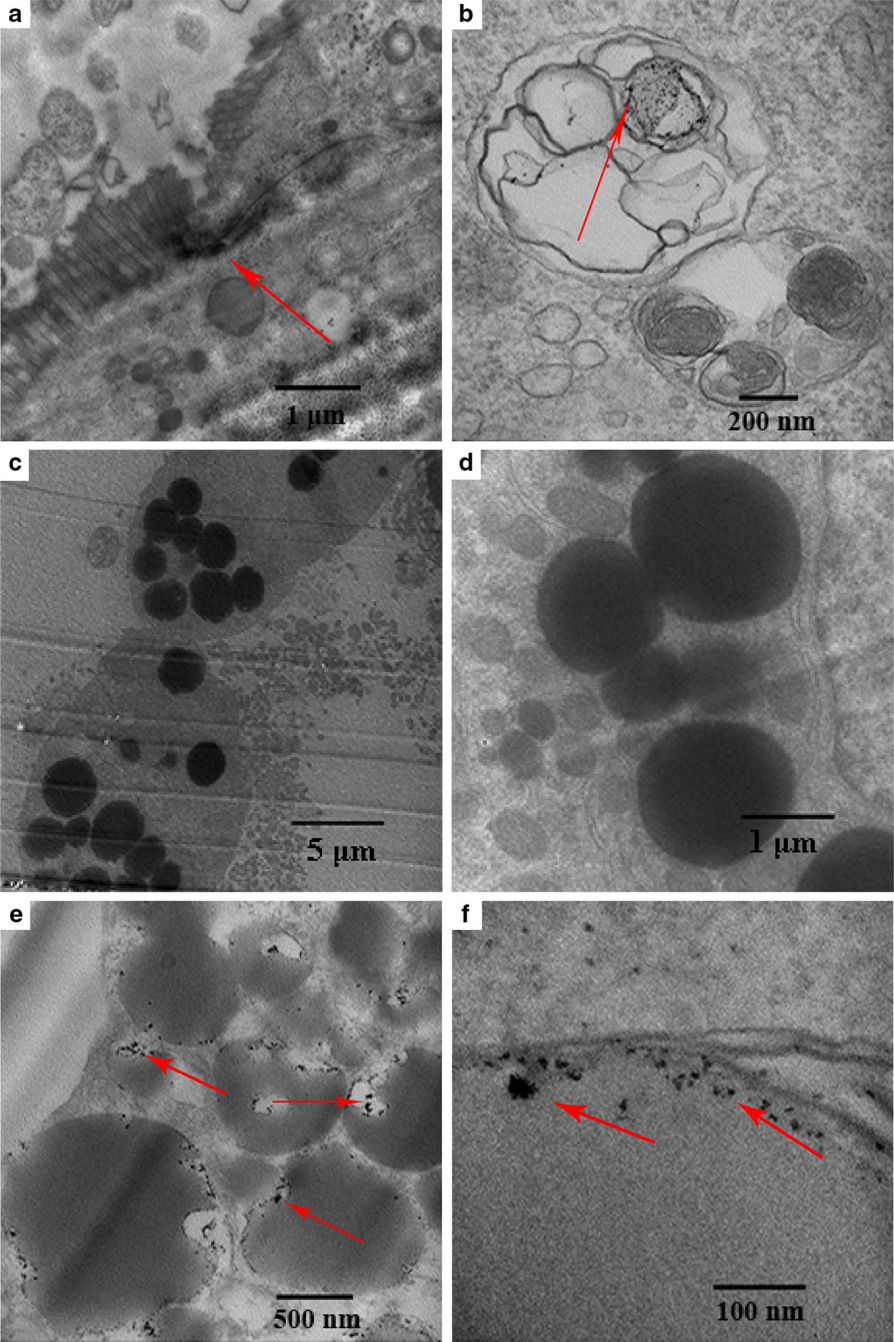
PPB absorbed by Caco-2 cells under TEM. **a** Intestinal villi; **b** lysosome; **c** lower magnification image of the cells with PPB treatment; **d** lipid droplets in the cells without PPB treatment; **e** lipid droplets in the cells with PPB treatment; **f** further enlarged image of PPB fragments on the edge of lipid droplets

Many studies have shown that PPB are a functional part of cellular metabolism since they provide materials for cells. Caco-2 cells were prepared in ultrathin sections after cultivation with 10 μg/mL PPB for 30 min. Cell sections without staining were observed by TEM as shown in Fig. 6. Black material with high contrast are PPB while other organic substances can’t be observed without staining. The intestinal villi can be seen on Caco-2 cells in Fig. 6a, indicating the success of the epithelial cell model construction. Black irregular fragments of PPB seen in Fig. 6b can be observed in lysosomes. However, only some debris was observed, and whole PPB were not observed in the cell interior. The possible reason for this phenomenon was that the PPB treatment time was a bit long. The capture of whole PPB form in cells is particularly difficult since PPB can be quickly absorbed and degraded in lysosomes. A lot of lipid droplets were observed in the cells cultivated with PPB under lower magnification (Fig. 6c). As the basic structure of PPB is polyP, PPB are lipophilic materials containing plenty of phosphate. Perhaps, cells can utilize PPB for their rich phosphate content to produce phospholipids with the result being an increased number of lipid droplets. The degraded PPB shown as Fig. 6e are mainly scattered around the edge of lipid droplets in cells, demonstrating that they have a strong lipophilic feature. Meanwhile, as we can see in Fig. 6d, no black debris were found around the lipid droplets in the cells without PPB treatment. On the further enlarged image (Fig. 6f), PPB can be observed very clearly distributed around the lipid droplets.

## Discussion

Here, we obtained almost double the yield of PPB through our transgenic method based on the observed fluorescence intensity. The yield of PPB can be affected by the nutrients in the medium, especially phosphorus. The yield of PPB may be improved further by optimization of culture conditions. The *ppk* gene studied in this paper, was obtained from *Synechococcus* sp. PCC 7002; thus, there is no need to worry about the safety of PPB from the *ppk*-type strain. We concluded that the engineered *Synechococcus* sp. PCC 7002 is a good “cell factory” for PPB high-efficient production.

From our data, we also concluded that PPB from *Synechococcus* sp. PCC 7002 are mainly distributed around the cell edge (Fig. 4). Unlike PPB from other *Synechococcus*, such as *Anacystis nidulan* (approximately 200 nm) [40], thermophilic *Synechococcus* sp. from microbial mats (approximately 100–300 nm) [2] and *S. elongatus* PCC 7942 (66.8–245.8 nm) [41], PPB from *Synechococcus* sp. PCC 7002 were smaller. The sizes of most PPB were below 100 nm (Fig. 5) and changed with the growth of cells. The PPB may be very small, just a few nanometres, at the beginning phase of cultivation and become larger in the later phases of growth. The PPB are also affected by environmental factors, especially the phosphorus content in medium. It seemed that PPB were much larger when given enough phosphorus than under phosphorus limited conditions [42]. Along with currently known uses, PPB may be developed into a new functionally active substances in the future.

In addition, PPB can be absorbed by Caco-2 cells effectively (Fig. 6), demonstrating that they can be absorbed in the mammalian intestine. Similarly, Segawa et al. [17] reported that polyP from *L. brevis* SBC8803 enhanced F-actin stability of human colonic epithelial Caco2/BBE cells and improved intestinal injury and survival rate of mice treated with a lethal dose of DSS. It may be possible that exogenous addition of PPB enhances the function of cells and improves the overall body function. Unlike the research from Segawa et al. [17] and Kashima et al. [18], we studied the absorption of the whole PPB not polyP in Caco-2 cells. Nanosized PPB without digestion may have better function with mineral elements retained.

In previous research, the overexpression of PPB improved cell tolerance to heavy metals [43–45] since PPB can help cells to store metal elements such as calcium, iron and zinc. Mineral substances entering cells with PPB may not easy be lost because of the lipophilic features. Thus, PPB may have a good absorption efficiency as a mineral supplement. As microalgae has a strong metal adsorption characteristic [46, 47], we can increase the concentration of mineral elements in culture medium to strengthen its absorbability and subsequently increase the mineral elements content in PPB. This observation provided an innovative idea for nutrient enrichment by biomineralization.

Microorganisms are one of the most attractive and simple sources for the synthesis of different types of metal nanoparticles [48]. Microalgae is rich in nutrition [49, 50], such as pigments, proteins, lipids, carbohydrates, vitamins and anti-oxidants, with applications in cosmetics, nutritional and pharmaceuticals industries [51]. *Synechococcus* plays an important role in the food cycle in the ocean. *Synechococcus* is a good source of nutrition, functioning as a primary producer. Marine active substances usually have a better effect than do terrestrial ones [52]. The effective utilization of marine PPB will improve the value of *Synechococcus* sp. PCC 7002. The yield improvement of PPB makes possible widespread use of PPB, and the specific composition and function of PPB is a question deserving further study.

## Conclusion

In the present study, we genetically engineered a *Synechococcus* sp. PCC 7002 strain that produced a twofold increase in PPB. The PPB were nanosized and ranged from a few to approximately 100 nanometres in diameter. Moreover, PPB were able to be effectively absorbed by Caco-2 cells and were predominantly scattered on the edge of lipid droplets. This article provides a promising “cell factory” for PPB production using engineering microalgae.

## Abbreviations

PPB: polyphosphate bodies
TEM: transmission electron microscopy
DLS: dynamic light scattering
DPAI: 4′,6-diamidino-2-phenylindole
RBS: ribosome binding site
PCR: polymerase chain reaction
PAGE: polyacrylamide gel electrophoresis
MEM: minimum essential medium
